# PUREAIR protocol: randomized controlled trial of intensive pulmonary rehabilitation versus standard care in patients undergoing surgical resection for lung cancer

**DOI:** 10.1186/s12885-017-3479-y

**Published:** 2017-07-31

**Authors:** Stefania Fugazzaro, Stefania Costi, Carlotta Mainini, Besa Kopliku, Cristian Rapicetta, Roberto Piro, Roberta Bardelli, Patricia Filipa Sobral Rebelo, Carla Galeone, Giorgio Sgarbi, Filippo Lococo, Massimiliano Paci, Tommaso Ricchetti, Silvio Cavuto, Domenico Franco Merlo, Sara Tenconi

**Affiliations:** 1 Physical Medicine and Rehabilitation Unit - Arcispedale Santa Maria Nuova-IRCCS, Viale Risorgimento 80, 42123 Reggio Emilia, Italy; 2 Department of Surgery, Medicine, Dentistry and Morphological Sciences, University of Modena and Reggio Emilia, Via del Pozzo n°71, 41124 Modena, Italy; 3 Department of Neuroscience, Rehabilitation, Ophthalmology, Genetics and Maternal Child Health, University of Genoa, L.go P. Daneo n°3, 16132 Genoa, Italy; 4 Unit of Thoracic Surgery - Arcispedale Santa Maria Nuova-IRCCS, Viale Risorgimento 80, 42123 Reggio Emilia, Italy; 5 Pulmonology Unit - Arcispedale Santa Maria Nuova-IRCCS, Viale Risorgimento 80, 42123 Reggio Emilia, Italy; 6 Research and Statistics Infrastructure Unit, Arcispedale Santa Maria Nuova-IRCCS, Viale Umberto I n°50, 42123 Reggio Emilia, Italy

**Keywords:** Rehabilitation, Lung neoplasms, Exercise therapy, Patient education, Breathing exercises, Exercise tolerance, Quality of life, Patient compliance

## Abstract

**Background:**

Non-small cell lung cancer is the most common type of lung cancer. Surgery is proven to be the most effective treatment in early stages, despite its potential impact on quality of life. Pulmonary rehabilitation, either before or after surgery, is associated with reduced morbidity related symptoms and improved exercise capacity, lung function and quality of life.

**Methods:**

We describe the study protocol for the open-label randomized controlled trial we are conducting on patients affected by primary lung cancer (stages I-II) eligible for surgical treatment. The control group receives standard care consisting in one educational session before surgery and early inpatient postoperative physiotherapy. The treatment group receives, in addition to standard care, intensive rehabilitation involving 14 preoperative sessions (6 outpatient and 8 home-based) and 39 postoperative sessions (15 outpatient and 24 home-based) with aerobic, resistance and respiratory training, as well as scar massage and group bodyweight exercise training.

Assessments are performed at baseline, the day before surgery and one month and six months after surgery. The main outcome is the long-term exercise capacity measured with the Six-Minute Walk Test; short-term exercise capacity, lung function, postoperative morbidity, length of hospital stay, quality of life (Short Form 12), mood disturbances (Hospital Anxiety and Depression Scale) and pain (Numeric Rating Scale) are also recorded and analysed. Patient compliance and treatment-related side effects are also collected. Statistical analyses will be performed according to the intention-to-treat approach. T-test for independent samples will be used for continuous variables after assessment of normality of distribution. Chi-square test will be used for categorical variables. Expecting a 10% dropout rate, assuming α of 5% and power of 80%, we planned to enrol 140 patients to demonstrate a statistically significant difference of 25 m at Six-Minute Walk Test.

**Discussion:**

Pulmonary Resection and Intensive Rehabilitation study (PuReAIR) will contribute significantly in investigating the effects of perioperative rehabilitation on exercise capacity, symptoms, lung function and long-term outcomes in surgically treated lung cancer patients. This study protocol will facilitate interpretation of future results and wide application of evidence-based practice.

**Trial registration:**

ClinicalTrials.gov Registry n. NCT02405273 [31.03.2015].

## Background

Lung cancer accounts for a fifth of the total global burden of disability-adjusted life years due to cancer [1]. Non-small cell lung cancer (NSCLC) is the most prevalent type of all lung cancers [1], with surgical resection appearing to be the most effective treatment in early stages (Stages I and II) [2].

Although surgical resection results in higher survival rates [3], it is associated with significant morbidity, functional limitations and decreased quality of life (QoL) [4].

Long-term physical impairment is an important and undesirable consequence of surgery, limiting patients’ recovery to a greater extent than do other severe pulmonary complications, such as atelectasis, dyspnoea and pneumonia [2, 5]. Furthermore, chronic obstructive pulmonary disease (COPD) is frequent in lung cancer patients and is associated with increased postoperative morbidity and mortality [6–8].

Recent studies suggest that perioperative pulmonary rehabilitation (PR) programmes including exercise may improve exercise capacity, functional performance and QoL, both pre- and postoperatively [2, 9].

PR is a comprehensive intervention that includes exercise training (endurance and resistance), education and behaviour changes [10, 11]. PR is also part of the new integrated pathways for enhanced recovery, where it has shown to improve patient satisfaction and to reduce in-hospital stay [12].

Surgical candidates seem to benefit from preoperative PR. In COPD patients, preoperative PR is correlated with improvement in functional parameters, which in turn may increase resection rate and allow more extensive lung resections [3, 10].

It is well known that exercise training is associated with significant increase in peak oxygen consumption (VO2peak) [13–15], which has been confirmed as a strong independent predictor of perioperative or postoperative complications and overall long-term survival for individuals with NSCLC [11, 16]. Indeed, patients with reduced exercise tolerance and low VO2peak show poorer thoracic surgical outcomes [11, 16]. Therefore, preoperative PR could reduce symptoms and morbidity, shorten hospital stay and lower healthcare costs [17, 18].

Concerning postoperative PR, recent literature supports improvement in exercise capacity through physical training [5, 19], whereas there is not sufficient evidence supporting meaningful clinical changes in lung function [5, 9, 19, 20]. The effectiveness of postoperative PR on QoL is still controversial, although some studies suggest that exercise training could be advantageous for some domains of QoL [19, 20].

Both pre- and postoperative PR tailored to lung cancer patients has been demonstrated to be safe and feasible [21, 22], although research conducted in this field presents dissimilarities concerning type of intervention, intensity, setting and timing [2, 5].

An updated, extensive literature review on perioperative physical exercise interventions was conducted by our group to establish the most updated and evidence-based interventions for patients undergoing surgery for NSCLC [23]. Based on its results, the best available evidence supports the inclusion of high intensity aerobic training for upper and lower limbs (primarily cycling and/or walking) and respiratory exercise (primarily inspiratory muscle training) in the perioperative period. Postoperative programmes should also include strength training and balance training. Therapeutic interventions can be conducted in both outpatient and home-based settings. Although this review focused on the exercise components of rehabilitation, most treatment protocols also include routine physiotherapy with dyspnoea management and airway clearance techniques. With regards to the length of interventions, while the duration of the preoperative phase is strictly determined by the time of elective surgery, postoperative intervention ranges from 6 to 20 weeks.

In summary, systematic reviews conducted in this field advocate the value of both pre- and postoperative PR programmes, with aerobic training as the most relevant component. However, this literature demonstrates that primary research validity has been limited by poor methodological quality and small and heterogeneous samples of clinical studies [2, 5, 23]. Thus, there is the need to build stronger evidence regarding the effectiveness of evidence-based PR interventions in this population.

### Rationale

The literature review provided sufficient evidence to justify a clinical trial to investigate the effects of pre- and postoperative intensive rehabilitation in patients affected by NSCLC undergoing surgical resection. Pulmonary Resection and Intensive Rehabilitation (PuReAIR) randomized controlled trial is designed to assess the effect of intensive PR on improving exercise capacity measured with the Six-Minute Walk Test (6MWT) in such patients.

The effects of intensive PR on postoperative morbidity, length of hospital stay (LoS), QoL, pain, depression and lung function will also be recorded.

The study protocol provides a detailed description of the interventions to accurately interpret results, which will be published on completion.

### Study objectives

#### Primary aim

The primary aim of this study is to investigate the effectiveness of intensive PR on exercise capacity in surgically treated NSCLC patients six months after surgery.

##### 1.2.1.1.Primary outcome measure

The primary outcome measure to assess the superiority of intensive PR over standard care (SC) is the change in distance walked in six minutes (6MWD) measured six months after surgery, compared to baseline.

#### Secondary aims

The secondary aims of this study are to investigate the effectiveness of intensive PR on:

1. Short-term exercise capacity
2. Lung function
3. Postoperative complications
4. Length of hospital stay
5. Quality of life
6. Mood disturbances
7. Pain

#### Secondary outcome measures

The secondary outcomes are:

1. Change in 6MWD measured one month after surgery and compared to baseline
2. Forced expiratory volume in 1 s (FEV1), forced vital capacity (FVC) and diffusing lung capacity for carbon monoxide (DLCO) measured six months after surgery and compared to baseline
3. Postoperative complications registered one month and six months after surgery as specific events
4. Interval (in days) between the date of the operation and the date of discharge
5. Change in Short Form 12 questionnaire (SF-12) scores measured six months after surgery and compared to baseline
6. Change in Hospital Anxiety and Depression Scale (HADS) score measured the day before surgery and six months after surgery, compared to baseline
7. Change in Numeric Rating Scale (NRS) score measured one month and six months after surgery, compared to baseline.

### Additional assessments

Data regarding patient compliance and treatment-related side effects during both preoperative and postoperative intensive PR will also be collected.

Intensive PR includes the implementation of hospital-based and home-based training. Patients randomized to the treatment group are requested to register the activities performed at home on a training log; distance in kilometres, number of steps and heart rate achieved during aerobic walking and exercises performed during respiratory muscle training (RMT) session are recorded. As patient adherence to protocol is a crucial issue in rehabilitation and the literature recommends the consistent and explicit reporting of exercise attendance in people with respiratory diseases [24], the proportion of training sessions performed are collected to assess patient compliance: adherence to the protocol is defined as the achievement of 80% or more of the training exercises prescribed [25].

We consider as minor side effects self-reported pain or discomfort in the muscles/joints involved with the physical component of the treatment; major side effects include any trauma secondary to a fall requiring specific interventions or symptomatic alterations of the basic vital signs (i.e., blood pressure, heart rate) arising during or shortly after the end of exercise and confirmed by instrumental measurement.

To evaluate the actual effect of lung resection on the study population before the postoperative treatment, FEV1, FVC and DLCO are also registered in the experimental group one month after surgery and compared to baseline.

## Methods

### Study design

A single institution 1:1 randomized controlled open-label trial with two parallel arms, powered for superiority, has been designed in accordance with the CONSORT statement and Helsinki declaration. The study has been approved by the local Ethical committee [n. 2013/0009390] and funded by the Italian Ministry of Health [GR-2011-02351711].

### Participants and setting

All patients with highly suspicious or diagnosed primary NSCLC are screened for eligibility criteria within the Lung Cancer Multi-Disciplinary Team Meeting at Arcispedale Santa Maria Nuova (ASMN) of Reggio Emilia, Italy. This is an Institute for Advanced Technologies and Healthcare Protocols in Oncological Research certified by the Organization of European Cancer Institutes in 2014. The Thoracic Surgery Unit’s catchment area covers a range of about 535,000 inhabitants and performs an average of 500 operations per year, including about 130 lung resections for malignancies.

Patients with Stages I and II NSCLC are considered for enrolment if they qualify for surgery. We exclude candidates who require adjuvant therapies and patients unfit for the physical exercise required by intervention [Table 1]. In patients affected by COPD, medical treatment is reviewed and optimized according to the most recent Global Initiative for Chronic Obstructive Lung Disease guidelines at the start of the selection process, to minimize confounding factors.

**Table 1:** Inclusion and exclusion criteria

Inclusion criteria	Exclusion criteria
Patients affected by NSCLC in Stage I-II	Tumour stage requiring adjuvant therapies
Patients eligible for lung resection	Patients unfit for the type of physical exercise included in study protocol
Patients able to walk autonomously without medical devices (e.g., crutches)	Patients affected by sensorial or cognitive deficits with potential severe impact on compliance (deafness, blindness, dementia, etc.)
Patients able to give informed consent	

**Fig. 1:**
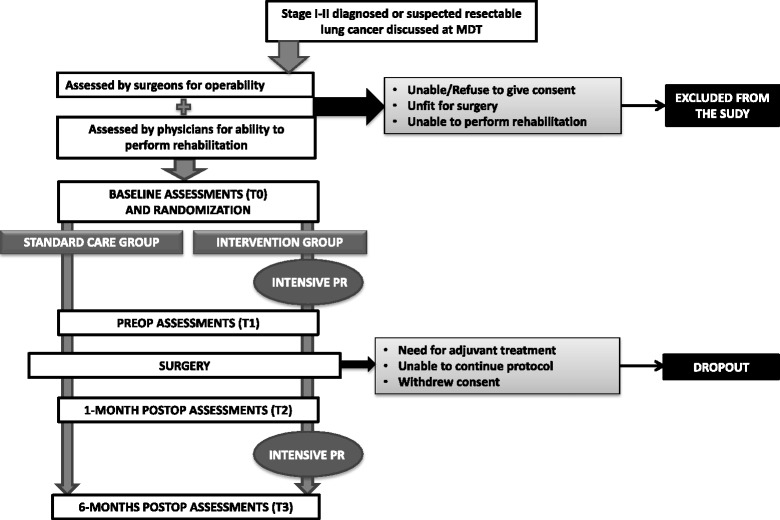
Study Flow diagram. Legend: MDT = Multi-Disciplinary Team; PR = pulmonary rehabilitation; Preop = preoperative; Postop = postoperative

Written informed consent is obtained from participants by the operating surgeon during the preoperative consultation. Subsequently, patients undergo baseline assessment and afterwards are randomized to receive intensive PR (Intervention Group - IG) or SC (Control Group - CG) (Fig. 1).

### Measurements

Patients included in the study are assessed at baseline (T0), the day before surgery (T1), one month after surgery (T2) and six months after surgery (T3) [Table 2].

**Table 2:** Assessments

	T0 Baseline	Randomization	T1 1 day before surgery (14÷21 days from baseline)		T2 (1 month ± 5 days after surgery)		T3 (6 months ± 5 days after surgery)	
			IG	CG	IG	CG	IG	CG
Pulmonary function (PFTs)	x		x				x	x
Exercise capacity (6MWT)	x				x	x	x	x
Quality of life (SF-12)	x						x	x
Mood disturbances (HADS)	x	Randomization	x	x			x	x
Pain (NRS)	x				x	x	x	x
Length of stay					x	x		
Postoperative complications					x	x	x	x

*IG* Intervention Group, *CG* Control Group, *PFTs* Pulmonary Function Tests, *6MWT* Six-Minute Walk Test, *SF-12* Short form 12, *HADS* Hospital Anxiety and Depression Scale, *NRS* Numeric Rating Scale

Baseline assessments are carried out immediately before randomization (T0) and include lung function, exercise capacity, QoL, mood disturbances and pain.

Static and dynamic respiratory volumes and DLCO are measured with full pulmonary function tests (PFTs). Exercise capacity is evaluated with 6MWT, according to current guidelines [11, 26]. Data on QoL are assessed with SF-12 [27], mood disturbances are evaluated using the HADS [28] and pain is quantified using the NRS [29].

The day before surgery (T1), all patients are re-assessed for mood disturbances. PFTs are repeated in the IG; treatment-related side effects and patient adherence to the intensive preoperative PR are also recorded.

One month after surgery (T2), patients repeat exercise capacity and pain evaluation using the same tools employed at baseline (T0). Data regarding LoS, postoperative complications and 30-day mortality are also recorded at this stage. Postoperative complication categories include acute respiratory failure, cardiac failure (including myocardial infarction), surgical site infection (including pneumonia and bronco-pleural fistula), arrhythmias (including atrial fibrillation), thrombosis and pulmonary embolism, neurological impairment (including stroke) and other (specified).

Long-term follow-up takes place 6 months after surgery (T3) and includes the assessment of lung function, exercise capacity, QoL, mood disturbances, pain and late postoperative complications. In the IG, patient adherence to the intensive postoperative PR and side effects are also recorded at 6 months.

To tailor the intensity of training, at T0 and T2 (immediately before initiation of pre- and postoperative PR) patients in IG perform:

- Shuttle walking test, to determine initial intensity of aerobic training [30];

- 10 repetition maximum test, to determine initial load of resistance training [31].

### Treatment protocols

Patients randomized to CG are provided with the SC already in place in our hospital. Patients randomized to IG follow an evidence-based intensive PR programme developed by the research group and delivered in addition to SC.

#### Control group

Patients allocated to CG receive SC, which consists of one therapeutic education session delivered by physician and physiotherapist the day before surgery and early inpatient postoperative PR, delivered by physiotherapist. The therapeutic educational session lasts 30 to 40 min and involves counselling and self-care management. The aim of counselling is to prepare the patients for the postoperative course, emphasising breathing exercise and sputum clearance techniques, pain control strategies and self-care. More specifically, breathing exercises focus on diaphragmatic breathing to prevent or relieve discomfort (shortness of breath, anxiety, pain), deep breathing to better ventilate all lung lobes and clearance techniques (huffing). These techniques are explained and repeatedly performed until the patient has mastered them. Self-care management involves pain relief and postural advice, as well as mobility techniques and practical advice in order to better cope with post discharge issues which could arise at home.

From day 1 after surgery until discharge patients receive daily early inpatient postoperative PR, which includes breathing exercises and positive expiratory pressure training with PEP bottle (PEP training) for a duration of 30 to 40 min every day. Upon discharge, patients are advised to continue breathing exercises and maintain an active lifestyle.

#### Intervention group

Patients allocated to the IG receive SC plus intensive PR, which includes a total of 53 sessions (14 preoperative and 39 postoperative).

##### 2.4.2.1.Preoperative intensive PR

Preoperative intensive PR is organised into six outpatient sessions performed two to three times per week, plus eight home-based sessions performed three to four times per week. The overall duration of this treatment ranges from 14 to 21 days, starting the same day the patient is listed for surgery or immediately afterwards, to minimize the delay in offering surgical treatment and to avoid the 30-day breach, as recommended by the regional guidelines [32].

Each outpatient session lasts approximately two hours and consists of an individual, supervised and personalised combination of the following elements:

- Therapeutic education, whose contents are the same as those provided to CG. It is delivered during the first outpatient session and repeated, if necessary, during the course of the treatment.
- Aerobic training, which consists of 30 to 40 min on a cycle ergometer. This training is maintained at the intensity of 60–80% peak workload (previously determined with shuttle walking test) [30] and includes a 5-min warm up and a 5-min cool down. Within this range, the intensity of training is adapted to the patient’s tolerance.
- Resistance training for lower limbs (extensor muscle group), upper limbs (biceps, triceps, deltoids, latissimus dorsi, pectoralis) and abdominal wall. Each exercise is performed for two to three sessions of 10 repetitions at the maximal load (previously determined with the 10-repetition maximum test) [31];
- RMT which includes breathing pattern training, PEP training and inspiratory muscle training for at least 15 to 30 min. Inspiratory muscle training is performed by means of a pressure threshold device, with a load ≥30% of maximal predicted inspiratory pressure; intensity is adapted to the patient’s tolerance.

Each home-based session lasts approximately one hour and consists of an individual, unsupervised and personalised combination of RMT, performed twice a day, plus 30 min of aerobic walking at the intensity of 60–80% of maximal heart rate. Patients are given a portable pedometer and a heart rate monitor and are requested to record their adherence to home-based training in the training log, which collects kilometres, steps and heart rate achieved during aerobic walking and exercises performed during RMT session.

##### 2.4.2.2.Postoperative intensive PR

Postoperative PR starts one month after surgery and includes 39 sessions divided into 15 outpatient sessions performed twice a week and 24 home-based sessions performed three times per week. Overall, the postoperative PR programme lasts 8 weeks.

- Each outpatient session takes approximately two hours and 15 min and includes both aerobic and resistance training, following the same procedures described for preoperative PR, plus RMT for the first eight sessions. In addition, outpatient postoperative PR includes 10 min of scar massage during the first four weeks and group bodyweight exercise training, scheduled once a week for the entire duration of the programme.
- Each home-based session lasts approximately one hour and consists of an individual, unsupervised and personalised combination of RMT performed twice a day and 30 min of aerobic walking at the intensity of 60–80% of maximal heart rate, monitored by patients with the portable device provided. Once again, patients are requested to record their adherence to home-based training by the training log.

At the end of postoperative PR, patients are advised to continue aerobic training and RMT until the 6-month follow-up is completed (T3).

### Standardization of procedures

All other aspects of patient management, such as perioperative care, general anaesthetic, intraoperative airway management and ventilation settings, postoperative analgesia, perioperative care and discharge plans, are routinely performed according to the current institutional protocols.

In particular, analgesia is provided and reviewed by the anaesthesiologist team and involves the positioning of epidural catheter before induction. Naropine 500 mg + Morphine 20 mg are administered though epidural catheter for the first 48 h, starting at the end of surgical procedure, with paracetamol 1 g every eight hours and ketoprofen 1 fl intravenously. Epidural catheter is removed usually on the third day after drainage elimination and therapy is shifted per os until discharge. Pain killer reduction is started one week after discharge.

To ensure the highest level of standardization of both the administration of treatments and the evaluations, two physiotherapists have been specifically trained for the purpose of this study; they are also responsible for collecting data regarding the outcome measurements (i.e., 6MWT, SF-12, HADS, NRS).

### Withdrawal from trial

Participation in the study will be withdrawn if any of the following occurs:

- Patient referred for adjuvant treatment
- Patient lost to follow-up
- Patient withdrawal of consent
- Death of the patient

All withdrawals are recorded specifying the reason.

### Statistical considerations

#### Sample size

Since data for mean and standard deviations of 6MWT after lung surgery with or without treatment have never been published, Cohen’s medium effect size (d = 0.5) has been used to compute sample size based on the minimal significant variation reported (25 m) [33].

We also expect a potential dropout rate of about 10% for the aforementioned reasons. Thus, assuming 5% type I error and 80% power for this study, we plan to enrol 140 patients (about 70 patients in each arm).

#### Randomization procedure

Patient enrolment is performed by the study data managers or physicians with a phone call to the local Research and Statistics Unit, which generated the allocation sequence: the responder simultaneously enters the patient and caller names and uses a predefined randomization list to make the assignment. Group allocation is revealed to researchers performing interventions and to patients after baseline evaluations (T0) are completed.

#### Blinding

This is an open-label study, due to the limited number of professionals involved in a single institution project.

#### Statistical analysis

Statistical analyses will be performed according to the intention-to-treat approach. Statistical techniques per study aims are as follows:

- *Primary aim:* t-test for independent samples to compare the mean of the change (defined as T3 – T0 related date) in 6MWD between IG and CG. In case of heteroscedasticity, checked by folded F test, the Satterthwaite adjustment will be used.
- *Secondary aims*:
  - t-test for independent samples to compare the mean of the change between IG and CG for 6MWD (T2 – T0 change), FEV1, FVC, DLCO and SF-12 score (T3 – T0 change), HADS score (T1 - T0 and T3 - T0), NRS score (T2 – T0 and T3 – T0) and LoS. In case of heteroscedasticity, checked by folded F test, the Satterthwaite adjustment will be used.
  - chi-square test to compare the proportion of postoperative complications between IG and CG;

Five percent significance will be used to assess the *p*-values and 95% two-sided confidence interval will be provided for each tested parameter; the confidence intervals will be calculated assuming normal distribution.

Statistical calculations will be performed by the local Research and Statistics Unit using SAS System release 9.2 or later, R release 3.3.3 or later, SPSS release 23 or later, according to the availability at the time of the data analysis.

Patient adherence to protocol may be a crucial issue in determining the efficacy of rehabilitation programmes, including exercise training. Although it has not been plainly demonstrated, in principle, non-attendance could affect exercise dose and influence the outcome of treatment [34]. Therefore, if patient compliance, is less than 80% [25], we will proceed with a second per-protocol analysis.

#### Duration and timeline

The study started enrolling in 2015. Results are expected by the end of 2017.

## Discussion

The main aim of PuReAIR is to assess the effectiveness of intensive combined pre- and postoperative PR in improving exercise capacity, lung function and long-term outcomes (QoL, pain, anxiety and depression) in lung cancer patients surgically treated with curative intent.

So far, the long-term effect of physical therapy on exercise tolerance and quality of life has only been reported in small series, and recent reviews have highlighted the need for well-designed studies to collect stronger evidence and clarify the role of perioperative PR [2, 5, 23].

The RCT design adopted allows reducing possible sources of bias (selection bias) and confounding factors which could be embedded in the heterogeneity of the target population. Moreover, this study design was powered according to an a priori specified hypothesis of superiority of intensive PR compared to SC.

The intervention of any trial should always be based on the best available evidence and described in sufficient detail to be reproducible. The study protocol provides a detailed description of the interventions delivered; this is particularly important in trials focusing on complex interventions, as is the case in rehabilitation. In fact, a comprehensive description of both standard care and additional treatments allows an accurate interpretation of the conclusions and ensures the correct replication of the protocol described, if appropriate, in order to compare results.

The definition of the best PR treatment programme required a careful review of the current literature [23], resulting in the definition of the following key elements:

- The inclusion of both aerobic and strength training of lower and upper limb muscles [2, 11, 35];
- The combination of outpatient and home-based treatment sessions. This should facilitate the enrolment of patients referred from provincial hospitals and might increase their compliance, minimizing the risk of attrition bias;
- The inclusion of inspiratory muscle training, incentive spirometry, airway clearance techniques and/or respiratory exercises, as lung cancer leads to respiratory impairment and COPD is a frequent comorbidity in patients affected by lung cancer [19, 20, 36, 37];
- The inclusion of group exercise training sessions during the postoperative phase, alongside individual guided and monitored home-based training, to facilitate mutual support among patients with NSCLC, whose vulnerable psychosocial condition could affect postoperative functioning and QoL [38]. In fact, a recent systematic review examining the spectrum of health care needs among people with lung cancer showed that psychological, emotional and social needs were most frequently specified and lack of acknowledgement of patients’ status by others were perceived as important issues [39]. Therefore, group sessions may facilitate the sharing of feelings, experiences and concerns, and may support patients in coping with their illness;
- The inclusion of scar massage in the postoperative phase to relieve tissue tension and reduce pain. In our clinical experience, patients frequently report pain and hypersensitivity around surgical scar.

At the time of planning this protocol, we estimated an overall 10% withdrawal rate, mainly due to perioperative morbidity and mortality, as well as unforeseen N1/N2 lymph nodal involvement diagnosed after surgery and requiring adjuvant therapies. However, more recent literature suggests a greater incidence of intraoperative lymph nodal upstaging [40] and even when an accurate staging workup is performed, including PET/CT and EBUS-TBNA when appropriate, minimal involvement of hilar nodes (N1 stage) remains difficult to assess preoperatively. Furthermore, more aggressive protocols are recommended by international guidelines in treating N1 NSCLC [41]. A greater than expected number of patients may therefore withdraw post randomization to ensure they receive the best cancer care.

In addition, because preoperative diagnosis may not always be obtained and, whenever possible, it seems to be a significant barrier to trial enrolment [42], we chose to include patients without proven cancer histology, providing that the clinical evaluation is consistent with malignancy (PET positivity, history of smoking, radiological features of malignancy, absence of concomitant extra-thoracic malignancies).

The exclusion of patients undergoing adjuvant therapies might represent a limit of this study, because it leads to a further selection of patients that could impact reproducibility in the future. However, in patients with lung cancer little is known about feasibility of physical exercise during chemo or radiotherapy, whose side effects might offset the potential benefits of PR. Thus, we chose to sacrifice broader inclusion criteria to find reliable answers, first and foremost, in the population that could most benefit from PR.

We emphasize the need for a timely delivery of preoperative PR to comply with the regional guidelines, which recommend starting cancer treatments within 30 days from referral [32]. If the results of this study show a clear benefit of intensive PR in patients with lung cancer, especially in improving QoL, a future trial on combined PR and non-radical treatments to extend the benefit of improved functioning to patients with advanced disease may be warranted. Likewise, the use of preoperative PR, if beneficial, will need to be evaluated in patients with severe COPD to increase the rate of lung resection.

## Conclusion

Due to the growing interest in perioperative PR, we are confident that PuReAIR will contribute significantly to providing reliable recommendations for systematic application of PR to patients undergoing surgery for early stage lung cancer. Moreover, the hybrid intervention adopted (inpatient-outpatient and home-based, group and individual sessions) could facilitate adherence to the protocol, which is determined by a combination of personal and health-related factors and might be pivotal in the rehabilitation of individuals with NSCLC [43]. The results of PuReAIR will be disseminated and will help healthcare professionals in implementing effective and affordable strategies to improve the care of cancer patients.

## Abbreviations

6MWD: distance walked in six minutes
6MWT: Six-Minute Walk Test
ASMN: Arcispedale Santa Maria Nuova
CG: Control Group
CONSORT: Consolidated Standards of Reporting Trials
COPD: Chronic Obstructive Pulmonary Disease
DLCO: Diffusing Lung capacity for Carbon monoxide
EBUS-TBNA: Endobronchial Ultrasound Transbronchial Needle Aspiration
FEV1: Forced Expiratory Volume in 1 s
FVC: Forced Vital Capacity
HADS: Hospital Anxiety and Depression Scale
IG: Intervention Group
LoS: Length of hospital Stay
NRS: Numeric Rating Scale
NSCLC: Non-small cell lung cancer
PEP: Positive Expiratory Pressure
PET/CT: Positron Emission Tomography and Computed Tomography
PFTs: Pulmonary Function Tests
PR: Pulmonary Rehabilitation
PuReAIR: Pulmonary Resection and Intensive Rehabilitation study
QoL: Quality of Life
RMT: Respiratory Muscle Training
SC: Standard Care
SF-12: Short form 12
VO2peak: peak oxygen consumption
